# A novel wavelet decomposition and transformation convolutional neural network with data augmentation for breast cancer detection using digital mammogram

**DOI:** 10.1038/s41598-022-09905-3

**Published:** 2022-04-08

**Authors:** Olaide N. Oyelade, Absalom E. Ezugwu

**Affiliations:** 1 School of Mathematics, Statistics, and Computer Science, University of KwaZulu-Natal, King Edward Avenue, Pietermaritzburg Campus, Pietermaritzburg, 3201 KwaZulu-Natal South Africa; 2 Depratment of Computer Science, Ahmadu Bello University Zaria-Nigeria, Zaria, Nigeria

**Keywords:** Cancer, Engineering, Mathematics and computing

## Abstract

Research in deep learning (DL) has continued to provide significant solutions to the challenges of detecting breast cancer in digital images. Image preprocessing methods and architecture enhancement techniques have been proposed to improve the performance of DL models such as convolutional neural networks (CNNs). For instance, the wavelet decomposition function has been used for image feature extraction in CNNs due to its strong compactness. Additionally, CNN architectures have been optimized to improve the process of feature detection to support the classification process. However, these approaches still lack completeness, as no mechanism exists to discriminate features to be enhanced and features to be eliminated for feature enhancement. More so, no studies have approached the use of wavelet transform to restructure CNN architectures to improve the detection of discriminant features in digital mammography for increased classification accuracy. Therefore, this study addresses these problems through wavelet-CNN-wavelet architecture. The approach presented in this paper combines seam carving and wavelet decomposition algorithms for image preprocessing to find discriminative features. These features are passed as input to a CNN-wavelet structure that uses the new wavelet transformation function proposed in this paper. The CNN-wavelet architecture applied layers of wavelet transform and reduced feature maps to obtain features suggestive of abnormalities that support the classification process. Meanwhile, we synthesized image samples with architectural distortion using a generative adversarial network (GAN) model to argue for their training datasets' insufficiency. Experimentation of the proposed method was carried out using DDSM + CBIS and MIAS datasets. The results obtained showed that the new method improved the classification accuracy and lowered the loss function values. The study's findings demonstrate the usefulness of the wavelet transform function in restructuring CNN architectures for performance enhancement in detecting abnormalities leading to breast cancer in digital mammography.

## Introduction

Global statistics on breast cancer in 2021 showed that the disease remains the most diagnosed cancer among women. The study revealed that between 1990 and 2019, global breast cancer cases rose to 2,002,354 and recorded 700,660 deaths in 2019^1^. The burden associated with new cases and disease mortality is disturbing, as 33% and 81% of cases are in ages 30–49 and 30–59 years, respectively^2^. The increased survival rate from the disease is being corroborated by the need for early detection, sometimes using mammography. Considering the limitation of human experts in detecting subtle features suggesting early stages of the disease in several cases, computer-aided detection (CAD) systems such as deep learning models have been proposed^3^. Several studies have demonstrated good performance in the use of deep learning to increase detection rates and lower false-positive rates^4–10^. To further advance the use of deep learning, performance enhancement techniques such as image preprocessing, sample augmentation, and architecturally optimized deep learning models (such as CNNs), have been developed. While the image preprocessing technique is expected to enhance input samples, architectural improvement is targeted at increasing the detectability of features to aid the classification process. Wavelet decomposition is an image preprocessing method supporting feature enhancement through data transformation in images and holds high potential for improving CNN performances^11,12^. On the other hand, the use of convolutional operations to detect features from enhanced images often compliments nonlinear functions to support the exploitation of image samples^13^. A skillful combination of these feature enhancement and feature detection techniques supports classifying and detecting abnormalities in medical images.

Mammography plays a pivotal role in screening and diagnosing breast cancer in the early stages. Digitized versions of mammography images have been widely used as samples in deep learning models for experimentation. However, the shortage of radiologists with expertise in reading mammography images combined with perception error associated with interpreting images remains a challenge^14^. Additionally, the need to increase accuracy and lower high positive and negative rates has motivated the use of wavelet decomposition and some other image preprocessing methods^15^. For instance, segmentation and wavelet transform methods were combined in^16^ to enhance important features supporting feature detection. To discriminate between the features of heterogeneous and scattered densities in image samples^17^, applied the wavelet decomposition method with a coefficient of 1. A multiresolution wavelet decomposition method was proposed in^18^ to extract spectral features in image samples. Meanwhile, improving the feature detection process through an architectural adjustment to a CNN has also been researched. Using histopathology image inputs in^18^, the CNN structure was improved using a wavelet function to detect the spectral features in the samples to achieve accurate classification. To monitor the large-scale fluorochemical engineering process with high accuracy, a wavelet-CNN architecture was proposed in^19^. A 2D-CNN was restructured to accommodate the wavelet function to increase the multiresolution level and classification accuracy of hyperspectral image samples^20^. The inverse wavelet transform function in restructuring CNNs for image reconstruction was applied in^21^, yielding a god performance.

Similarly, an extended CNN architecture, coupled with wavelet prediction loss, texture loss, and full-image loss, was applied in^22^ to increase the resolution of the multiscale face. Features related to COVID-19 were extracted from lung images using a proposed wavelet-CNN architecture^23^. Wavelet transforms also have been integrated into CNN architectures to improve the multiresolution analysis capability of hybrid structures^24^. Improving the classification accuracy of MNIST image samples has been proposed using a wavelet-convolution-wavelet-NN. The convolutional and fully connected layers are driven by a wavelet transform^25^. Wavelet transform has also been used in CNN to achieve spectral analysis for texture classification^26^.

After a detailed review of wavelet decomposition and wavelet transform in feature enhancement and feature detection tasks related to deep learning models, we found some critical limitations with the existing methods while addressing the classification of digital mammography images. Although the wavelet decomposition operation can enhance features in image samples, it currently lacks provision to discern what features need enhancing and what features require elimination to optimize the feature enhancement process. Also, pixel coverage of some subtle abnormalities in real-life medical images may be substantially small, making it difficult for both human and vaguely implemented models to detect such anomalies. To address this gap, this study proposes a hybrid of seam carving and wavelet decomposition algorithms. The novel hybrid model is able to balance the optimization challenge between feature enhancement and elimination so that suggestive features are enhanced while non-relevant features are eliminated. Secondly, we reinforce our method to address further the challenge of extracting subtle features suggesting abnormalities through the convolutional operation. As a result, we propose a novel wavelet transform function suitable for addressing the problem of feature detection in a medical image. Meanwhile, we applied a generative adversarial network (GAN) model^27^ to generate image samples with architectural distortion abnormalities to augment the insufficient training data, leading to reducing a high false-positive rate that does not generalize^28^. In addition, samples were preprocessed to eliminate low contrast in the training datasets, which often impairs the performance of CNNs^29^. The technical contributions of this paper are highlighted below:

i. Design of a new CNN structure that uses a novel wavelet transform function
ii. Design of a hybrid algorithm of seam carving and wavelet decomposition to support feature enhancement in the image preprocessing phase.
iii. Incorporation of a new GAN model for image synthesis and augmentation in the proposed CNN model.
iv. Comparative analysis of the new method was validated using DDSM + CBIS and MIAS datasets.

The rest of the paper is organized as follows: “Related works” presents reviews on related studies. “Methodology” provides an overview and design of the concepts proposed in this paper. “Experimentation” presents the system configuration, parameter settings, and datasets used for experimentation. In “Results and discussion”, the results of the application of the proposed method are presented and discussed. Finally, in “Conclusion”, the conclusion of the paper is discussed.

## Related works

This section presents a review of some related works that used data augmented techniques for training deep learning models in detecting abnormalities from digital mammography and other related areas. Abnormalities in mammograms are often categorized into four categories: malignant mass, calcification, architectural distortion, and asymmetric of the breast. All studies reviewed were selected using this consideration of abnormalities, wavelet functions, and data augmentation using GANs.

Using existing and trained architectures helps fast-track the process of adapting networks for applicability to other problems. This was demonstrated by^30^, who used AlexNet and some segmentation techniques to classify and segment ROIs. The authors modified AlexNet for binary classification by introducing a support vector machine (SVM) classifier at the last fully connected layer. The approach also used a segmentation technique, namely, threshold- and region-based, to automate the process of ROI extraction. The method for the classification was based on applying SVM on mammography images from the digital database for screening mammography (DDSM) and the Curated Breast Imaging Subset of DDSM (CBIS-DDSM). The research successfully classified benign and malignant mass tumors in breast mammography images by obtaining an accuracy of 87.2% with an AUC equal to 0.94 (94%). Similarly, Levy and Jain^31^ investigated the performance of the following architectures: AlexNet, GoogLeNet, and a shallow CNN architecture. The three models were used for classifying images, whether malignant or benign, based on the detection of malignant masses. To circumvent the challenge of overfitting, they used transfer learning techniques, batch normalization, careful preprocessing, and data augmentation. For both AlexNet and GoogLeNet, the researchers used the same base architecture as the original works but replaced the last fully connected (FC) layer to output classes. The shallow CNN proposed takes a 224 × 224 × 3 image as input, and it consists of 3 convolutional blocks composed of 3 × 3, 3 fully connected layers, and a soft-max layer. Furthermore, they employed ReLU activation functions, Xavier weight initialization, and the Adam update rule with a base learning rate of 10^−3^ and batched size 64. The best model presents a result of 0.934 for recall at 0.924 for precision.

In related work, Jung et al.^32^ proposed the use of RetinaNet to detect mass in mammograms. They made the RetinaNet model use weights pretrained on GURO, training and testing on INbreast. They observed that using weights pretrained on datasets achieves similar performance as directly using datasets in the training phase. Experimental setups using the public dataset INbreast and the in-house dataset GURO showed that their model obtained a good performance of an average number of false positives of 0.34 and 0.03 when the confidence score was 0.95 in INbreast and GURO, respectively. Similarly, Agarwal et al.^33^ employed transfer learning to propose a patch-based CNN method for automated mass detection in full-field digital mammograms (FFDM). In addition, they investigated the performances of VGG16, ResNet50, and InceptionV3 architectures on the same dataset while applying the transfer learning technique to uncover the benefit of domain adaptation between the CBIS-DDSM (digitized) and INbreast (digital) datasets using the InceptionV3 CNN. Their experimentation showed that InceptionV3 performs best for classifying the mass and non-mass breast regions for CBIS-DDSM. The results show that transfer learning from CBIS-DDSM obtains a substantially higher performance with the best true positive rate (TPR) of 0.98 at 1.67 false positives per image (FPI) compared with transfer learning from ImageNet with a TPR of 0.91 at 2.1 FPI. In^34^, the authors demonstrated the existence of superiority when a deep learning-based classifier was used to distinguish malignant and benign breast masses without segmenting the lesions and extracting the predefined image features. In^35^, an adversarial deep structural network was adopted for use on mammographic images in detecting mass segmentation. The research employed a fully convolutional network (FCN) to model the potential function, followed by conditional random fields (CRF) to perform structural learning. This end-to-end model was used for mammographic mass segmentation. While combining FCN with position a priori for the classification task, GAN training was used to control overfitting due to the small size of mammogram datasets. Four models with different convolutional kernels were further fused to improve the segmentation task. The results showed that the end-to-end model combined with adversarial training achieves state-of-the-art performance on two public datasets: INbreast and DDSM-BCRP.

The work in^36^ combined Craniocaudal (CC) and Mediolateral-oblique (MLO) mammography views to differentiate between malignant and benign tumors. They implemented a deep-learning classification method that is based on two view-level decisions, implemented by two neural networks, followed by a single-neuron layer that combines the view-level decisions into a global decision that mimics the biopsy results. The model exploited the detection of features of clustered breast microcalcifications to classify tumors into benign and malignant categories. In related work, Sert et al.^37^ adapted a CNN model to the task of breast tumor classification as benign or malignant based on the detection of microcalcification features. Basically, the approach investigated the benefit of employing various preprocessing methods, such as contrast scaling, dilation, cropping, and decision fusion, using an ensemble of networks and the CNN model. Experimental results showed that preprocessing greatly improved classification performance. The learning models proposed achieved a recall of 94.0% and precision of 95.0% above human-level performance. Additionally, Xi et al.^38^ was able to use classifiers that are trained on labeled image patches and then adapted it to work on full mammogram images for localizing the abnormalities. The models investigated are VGGNet and ResNet, demonstrating the most appreciable accuracy at 92.53% in classifications. Meanwhile, Murali and Dinesh^39^ employed a deep convolutional neural network (CNN) and random forest classifier to classify ROIs with malignant masses and microcalcifications. The AUC of the CNN was 0.87, which was higher than the radiologists' mean AUC (0.84), although the difference was not significant. On the other hand, the studies discussed in^40,41^ circumvent the use of deep learning by adopting wavelet decomposition.

A recent study^5^ proposed combining CNN architecture with image augmentation to detect architectural distortion. Many transformation operations were applied to the image samples with right and left breasts presented in MLO and CC views for augmentation purposes. The resulting model was applied to ROIs from MIAS, whole images from INbreast, whole images from MIAS, and ROIs from DDSM + CBIS databases. Performance evaluation of the proposed model showed that they achieved an accuracy of 93.75%. The use of Region-based (R-CNN) was introduced in^42^ to detect architectural distortion using a supervised pretrained region-based network (R-CNN). Experimentation was based on the DDSM dataset, and the results showed that they obtained over 80% sensitivity and specificity and yielded 0.46 false positives per image at an 83% true-positive rate. Similarly, the work in^43^ demonstrated a novel neural network that combined two learning branches with region-level classification and region ranking in weakly and semisupervised settings. Their results for weakly supervised learning showed an improvement of 4% in AUC, 10–17% in partial AUC, and 8–15% in specificity at 0.85 sensitivity. On the other hand^35^, GlimpseNet autonomously extracts multiple regions of interest, classifies them, and then pools them to obtain a diagnosis for the full image. They obtained the result that gained 4.1% improvement. Additionally, Qiu et al.^44^ proposed a framework using a deep convolutional neural network. The model is an 8-layer deep learning network that involves 3 pairs of convolution-max-pooling layers for automatic feature extraction and a multiple layer perceptron (MLP) classifier for feature categorization to process ROIs. The MLP classifier comprises one hidden layer and one logistic regression layer. The results of their experimentation achieved AUCs of 0.696 ± 0.044, 0.802 ± 0.037, 0.836 ± 0.036, and 0.822 ± 0.035 for fold 1 to 4 testing datasets, respectively, with an overall AUC of 0.790 ± 0.019 for the entire dataset. In another related work, Bakkour and Afdel^45^ proposed a novel discriminative objective for a supervised feature deep learning approach focused on classifying tumors in mammograms as malignant or benign, using the Softmax layer as a classifier. The proposed network was enhanced with a scaling process based on Gaussian pyramids to obtain normalized size regions of interest. The DDSM and BCDR datasets were used in addition to the data augmentation technique. The results of their experiments showed that they obtained an accuracy of 97.28%.

In^46^, the authors presented a novel classification technique for a large data set of mammograms using deep learning: convolutional neural network-discrete wavelet (CNN-DW) and convolutional neural network-curvelet transform (CNN-CT). An augmented data set is generated by using mammogram patches and filtering the data, by contrast, limited adaptive histogram equalization (CLAHE). At the same time, the softmax layer and support vector machine layer were used as classifiers. The results showed that CNN-DW and CNN-CT achieved accuracy rates of 81.83% and 83.74%, respectively. The authors in^47^ used a wavelet convolution neural network to detect spiculated findings in low-contrast noisy mammograms, such as architectural distortions and spiculated masses. The dataset used for experimentation consisted of CBIS-DDSM, and it reached an accuracy of over 85% for architectural distortions and 88% for spiculated masses. The databases used are the IRMA version of the digital database for screening mammograms (DDSM) and the Mammographic Image Analysis Society (MIAS). The results pertain to an accuracy of 92.94% obtained in the case of the DDSM database for fixed-size ROIs and for the MIAS database, an accuracy of 95.34%. Other studies that have used similar approaches, although with application in different domains, are as follows: the use of wavelet convolutional neural network (wCNN) and wavelet convolutional wavelet neural network (wCwNN) for image classification on MNIST dataset^48^, and the use of wavelet function for feature extraction to support CNN-based feature detection in the classification of lung cancer using computerized tomography (CT) scans^12^.

In addition to using wavelet-based CNN in medical image classification, several domains have also received attention in applying the technique. For example, Peifeng et al.^49^ proposed integrating a wavelet function on time series data and into a backpropagation neural network (BPNN) and nonlinear autoregressive network with exogenous inputs (NARX) to achieve WNN and WNARX hybrid models, which were applied as benchmark models. Experimentation with the hybrid model showed that the wavelet transform could enhance long-term concentration predictions. In another novel approach, Nourani et al.^50^ applied the wavelet function to a variant of the SVM to obtain a Wavelet-based Least Square Support Vector Machine (WLSSVM) model. The study then used the WLSSVM to predict Suspended Sediment Load (SSL) in a river. Meanwhile, an artificial neural network (ANN) was adapted for feature extraction to support the WLSSVM model. In another study, Gürsoy et al.^51^ attempted to predict the actual discharge using meteorological data based on a wavelet neural network method. Wang et al.^52^ analyzed, classified, and forecasted time series data for frequency-awareness using a multilevel Wavelet Decomposition Network (mWDN) supported by Residual Classification Flow (RCF) and multi-frequency Long Short-Term Memory (mLSTM) deep learning models. In a similar domain, Wuwei et al.^53^ investigated the use of both wavelet neural network and data fusion models. Meanwhile, an RBF algorithm and SPSS Clementine technique were also combined to support the wavelet transform sequences for the prediction process. Shah et al.^54^ forecast output growth using wavelet transforms and Levenberg–Marquardt (LM) ANN models.

We now present a summary of all related works and compare their methods with that which is proposed in this study. Existing methods and techniques in literature used to address the problem motivating this study still present some gaps justifying the need for improvement. As reported by^30^, the use of ROIs does not address the need for feature enhancement in the ROIs samples. Moreover, the ReLU activation function in^31^ still generalizes on a well-known activation function. Also, using two deep learning models in^36^ for feature detection is computationally costly compared with the one-model feature-detection-enhancement inclusive mechanism proposed in the model presented in this paper. Similarly, the use of only mainstream preprocessing techniques has no guarantee that relevant features can be isolated and enhanced. As such, the approach in^37^ lags behind what is proposed in this study. The popularity of the R-CNN method as used in^42^ for region-level abnormality detection still suffers from the omission of sensitive features owing to their automated region selection algorithm. A similar approach in^43^ leaves out the use of an optimized method for selecting regions in the second branch of their dual-branch model. We found our proposed method competitive with what is reported in^46,48^ so that performance obtained based on the variation of both methods put this study ahead of^46,48^.

## Methodology

This section presents the proposed concept, which describes the application of seam carving and wavelet decomposition techniques to feature enhancement and extraction of CNN architectures. First, we discuss the design encompassing the model, which subsumes other methods used in the study. Next, the details of the GAN architectures used for image synthesis are presented. Additionally, a detailed presentation using the mathematical formulation was used to discuss the image preprocessing and preparation techniques used. Finally, we present the design of the proposed CNN and wCNN architectures.

### Overview of methodology

The following are the procedures that outline the overview of our approach:

- Images are extracted from records representation in the DDSM + CBS database to PNG representation for storage on the file system
- A GAN model trained in^27^ is applied for the image synthetization process to augment the class imbalance in the extracted image samples. The synthetization is necessitated by the need to allow the deep learning model to generalize well on all classes of image samples.
- A combination of the images drawn from the real and synthesized distributions are then applied to an image enhancement technique, namely, contrast-limited adaptive histogram equalization (CLAHE).
- The preprocessed image samples from step (c) are applied to the seam carving algorithm to remove low-energy pixels.
- The wavelet decomposition packet function is then used to extract a high resolution and rich feature representation of each image sample output obtained from the seam carving procedure. All samples processed using this procedure are passed into the feature extraction and classification step.
- To investigate and compare the performance of the traditional CNN and the proposed wCNN, we supplied the processed images to them for a complete training phase.
- The trained CNN and wCNN architectures are then tested on the test dataset for evaluation using selected metrics.
- The results are then compared for discussion on findings from the study.

**Figure 1:**
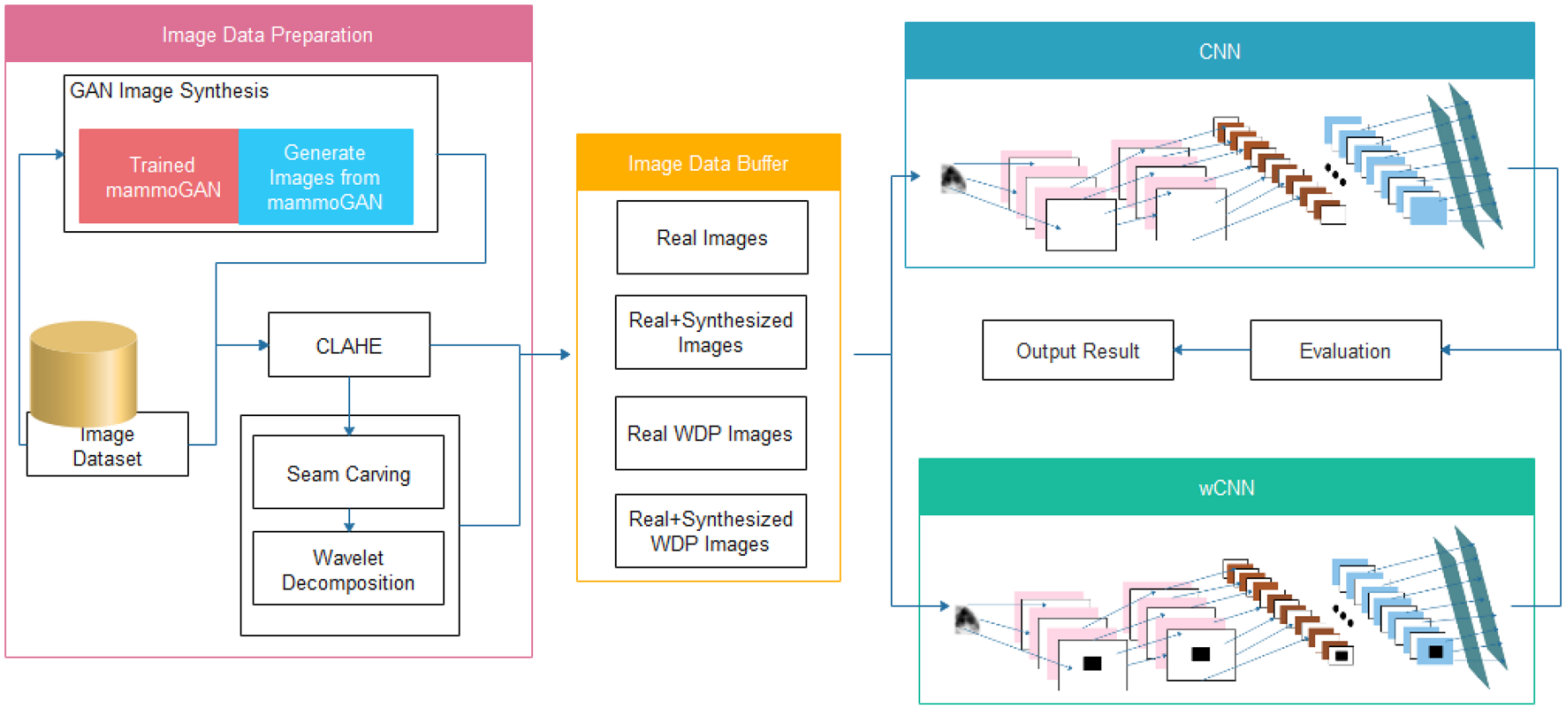
Block diagram describing the overview of the approach used in this study, which consists of image preprocessing, GAN-based augmentation, seam carving, wavelet decomposition, and wavelet convolution (CNN and wCNN) architectures.

In Fig. 1, an illustration of the overview of the approach outlined above is presented. The block diagram highlights the flow of the methods applied to achieve the study's aim. The remaining subsections are dedicated to describing each method and how it applies to the overall interest of the study.

### GAN architecture for image synthesis

**Figure 2:**
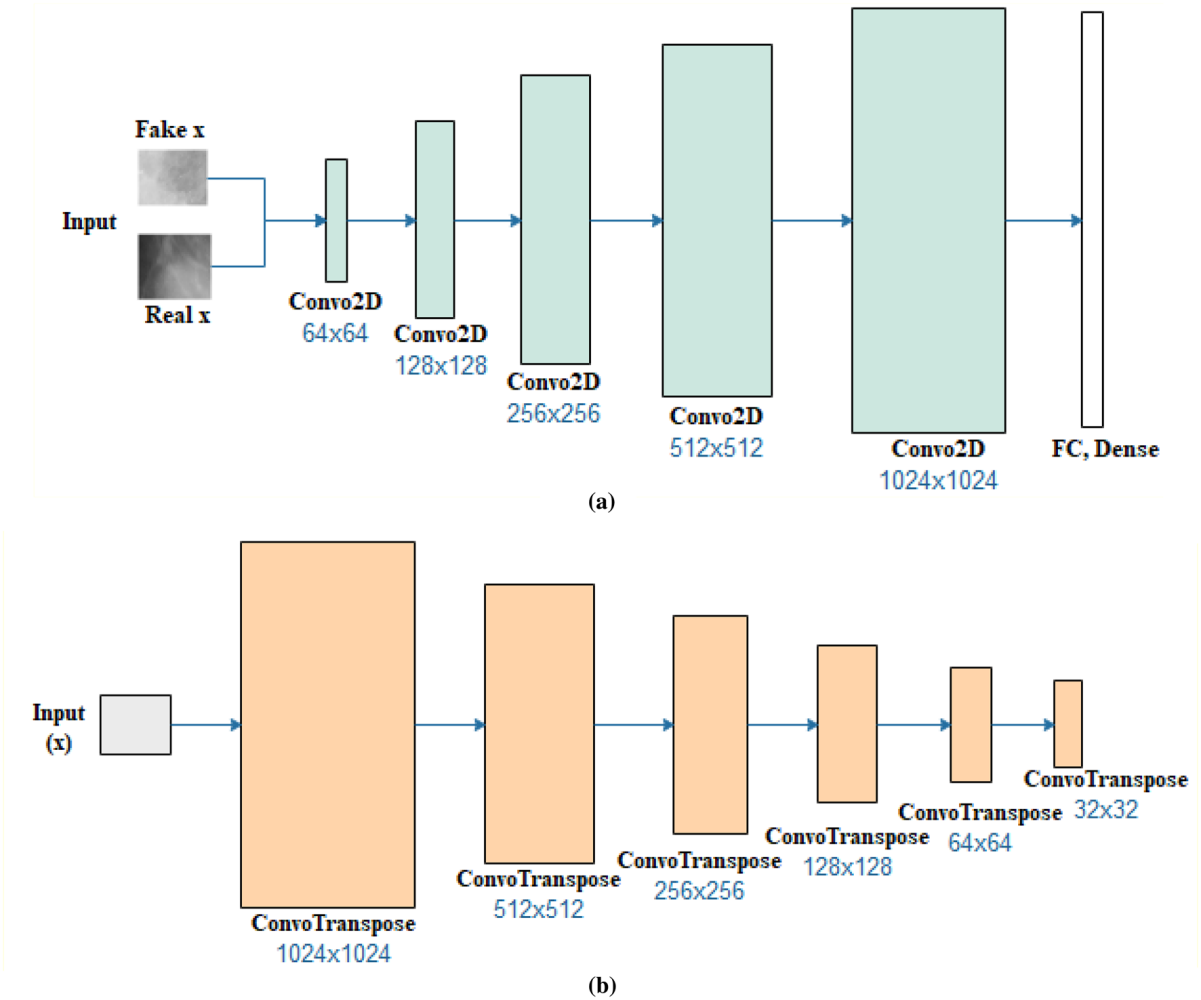
An illustration of the applied GAN model consisting of the (**a**) discriminator and (**b**) generator networks.

An adversarial architecture consisting of a generator and discriminator was applied for synthesizing image samples in this study. This became necessary to eliminate the perceived class imbalance observed in the dataset used for the experimentation. Moreover, we adapt this data augmentation technique to strengthen further the performance of the deep learning model proposed in the study. We considered the high impact of image synthesis over image transformation, which are both types of data augmentation techniques, to enhance the performance and balance the class distribution of samples in our dataset. A detailed representation of the GAN model applied for the image synthesis task is described in Tables 1 and 2. A further illustration of the two architectures represented by the discriminator network, D and generator network, G are captured in Fig. 2a,b, respectively.

**Table 1:** Generator architecture: we adopted the input noise vector of dimensionality 100 drawn from a zero-mean Gaussian distribution.

	Input Projection	Layer1	Layer2	Layer3	Layer4	Layer5	Layer6
Type	Fully Connected	Fractionally Strided Convolution	Fractionally Strided Convolution	Fractionally Strided Convolution	Fractionally Strided Convolution	Fractionally Strided Convolution	Fractionally Strided Convolution
Input	[1 × 100]	[4 × 4 × 1024]	[8 × 8 × 512]	[16 × 16 × 256]	[32 × 32 × 128]	[64 × 64 × 64]	[128 × 128 × 32]
Output	[4 × 4 × 1024]	[8 × 8 × 512]	[16 × 16 × 256]	[32 × 32 × 128]	[64 × 64 × 64]	[128 × 128 × 32]	[64 × 64 × 2]
Activation	ReLU	ReLU	ReLU	ReLU	ReLU	ReLU	TanH
Batch Norm	Yes	Yes	Yes	Yes	Yes	Yes	Yes
Stride	–	2	2	2	2	1	-
Padding	–	Same	Same	Same	Same	Same	Same
Kernel Size	–	5	5	5	5	5	5
Kernels	–	1024	512	256	128	64	32

Minibatch size: 32, optimizer: adaptive moment estimation (Adam) (η = 0.00001, β1 = 0.5, β2 = 0.999). All weights were initialized using the normal distribution initializer.

**Table 2:** Discriminator architecture: minibatch size: 32; optimizer: Adam (η = 0.0001, β1 = 0.5, β2 = 0.999).

	Layer1	Layer2	Layer3	Layer4	Layer5	Output
Type	Convolution	Convolution	Convolution	Convolution	Convolution	Full Con
Input	[32 × 32 × 2]	[64 × 64 × 64]	[32 × 32 × 128]	[16 × 16 × 256]	[8 × 8 × 512]	[4 × 4 × 1024]
Output	[64 × 64 × 64]	[32 × 32 × 128]	[16 × 16 × 256]	[8 × 8 × 512]	[4 × 4 × 1024]	^1^
Activation	LeakyReLU	LeakyReLU	LeakyReLU	LeakyReLU	LeakyReLU	Sigmoid
Batch norm	Yes	Yes	Yes	Yes	Yes	–
Stride	2	2	2	1	1	–
Padding	Same	Same	Same	Same	Same	–
Kernel size	5	5	5	5	5	–
Kernels	64	128	256	512	1024	–

The discriminator network D consists of feature extractor *F* (*img*) and a layer for classification using a sigmoid function with weight vector ψ_*l*_. It also consists of five (5) fractionally strided convolution layers and the fourth layer of dense/flattened and fully connected layers that uses a sigmoid activation function. To overcome the problem of poor parameter initialization, batch normalization is performed on each of the layers except for the last layer. Each layer uses a kernel size of 5 × 5 and filter sizes of 64, 128, 256, 512, and 1024 with leaky rectified linear unit functions applied for the activation computation. On the other hand, the generator G consists of a fully connected layer projecting input of a 100-dimensional uniform distribution to six (6) fractionally strided convolutions having batch normalization applied, with filter sizes of 1024, 512, 256, 128, and 64, kernel size of 5 × 5, and rectified linear unit activation functions for each unit. The trained GAN model was then applied to synthesize images with different abnormalities associated with breast cancer in digital mammography. We then combined the synthesized images and the real samples for the image preprocessing method.

### Image preprocessing

Common image preprocessing involves color normalization, noise reduction, edge detection and histogram equalization. In this study, we prepared image samples for the CNN architecture by applying some image preprocessing techniques on samples for the purpose of histogram equalization and noise removal. That is, the noise was removed from breast image contrast enhancement and image breast segmentation to remove background area, labels, artefacts, and pectoral muscle. This paper applies contrast-limited adaptive histogram equalization (CLAHE) to improve the contrast in images. This procedure provides high-quality image samples and enhances the features in the samples for effective feature extraction.

In image preprocessing, image compression, separation and decrease are essential operations. One of the benefits of the compression operation is to allow for the removal of pixel(s) that have no significant information in the image and provide a multiresolution and high-resolution presentation of the image. This study combined two compression algorithms, namely, the seam carving and wavelet decomposition algorithms, to improve our image samples. Seam carving was applied to improve content awareness, thereby eliminating pixel(s) locations that, when removed, the image quality was not reduced, nor was the view distorted. The outcome of this is a resized image with no application of the cropping operation. The outcome of this is an image whose realism is preserved by parsing it top–bottom and left–right to identify optimal and suboptimal seams.

The approach for seam carving used in this study is first to compute the energy function or gradient matrix of our image samples using Eq. (1):

1 $${g}_{i,j}=\left|\frac{\partial Img}{\partial x} (i, j)\right|+ \left|\frac{\partial Img}{\partial y} (i, j)\right|$$

2 $$energy\;function\;of\;Img= {g}_{(x,y)}=\sum_{i=0}^{m-1}\sum_{j=0}^{n-1}\left|\frac{\partial Img}{\partial x} (i, j)\right|+ \left|\frac{\partial Img}{\partial y} (i, j)\right|$$

In Eq. (2), we use the energy function to obtain the total gradient matrix for an image, say *Img*. This provides us with information on the pixels to be preserved in both the horizontal and vertical directions of the image while the remaining pixels are removed. Once the seams of the sample images have been carved out, we passed the resulting images to the wavelet decomposition technique.

The second image compression and improvement technique applied is the wavelet decomposition packet. Using this technique, we can obtain high resolution and extract local spectral information of the output of the image from the seam carving operation. A few wavelet functions include haar, db1, db4, db16, coifi, sym4, sym8, bior1.3, and bior3.1, and we applied the haar function for this study to obtain the best output. Generally, the wavelet generating function can be expressed as in Eq. (3):

3 $$\Psi (a\cdot b)=\frac{1}{\sqrt{a}}\underset{-\infty }{\overset{\infty }{\int }}\stackrel{-}{\varphi \left(\frac{t-b}{a}\right)}Img\left(t\right)dt$$

4 $$a, b=\left\{\begin{array}{c}a={2}^{j}\\ b={2k}^{j}\end{array}\right.$$

where *a* is the scaling factor and *b* represents the shift factor, so that *a* and *b*, which control the extension and translation operations, are defined as shown in Eq. (4). Additionally, *Img(t)* denotes the representation of our image, and $\varphi$(t) represents the mother wavelet function, which is further described below. Now, given an image of size *Img(N,M)*, we show the 2D wavelet decomposition representation of the image as follows so that the wavelet function and the scaling function are represented using Eqs. (5) and (6):

5 $$\Psi \left(x,y\right)=\left\{\begin{array}{c}1\;\;  for\;\;  0 \le x,y\le 0.5\\ -1 \;\; for\;\;  0.5 \le x, y \le 1\\ 0\;\;  otherwise\end{array}\right.$$

6 $$\varnothing (x, y)=\left\{\begin{array}{c}1\;\;  for\;\;  0\le x, y \le 1\\ 0 \;\; otherwise\end{array}\right.$$

The decomposition of our image *Img* will yield four (4) coefficients, namely, LL, LH, HL, and HH, which are further categorized into the approximate coefficient (LL), also known as low pass, and the detailed coefficients (LH, HL, and HH), also known as high pass. LH, HL, and HH represent the horizontal (H) view of the details of the image, vertical (V) view of the details of the image, and diagonal (D) view of the details of the image, respectively. These four coefficients are mathematically computed using the following:

7 $$LL=\varnothing \left(x,y\right)=\varnothing (x)\varnothing (y)$$

Equation (7) gives the low pass scale function, and the representation of the corresponding scaling function is given in Eq. (8) as:

8 $${W}_{\varnothing }\left({s}_{0}, m, n\right)=\frac{1}{\sqrt{mn}}\sum_{x=0}^{m-1}\sum_{y=0}^{n-1}Img\left(x,y\right){\varnothing }_{{s}_{0}, m,n}(x,y)$$

where s_0_ represents the scale value and $m,n$ is the dimension of the image.

9 $$LH={\Psi }^{H}\left(x,y\right)=\Psi (x)\varnothing (y)$$

Similarly, Eqs. (9), (11), and (13) give the high pass scale functions for the H, V, and D wavelets, and the representation of the corresponding wavelet function is given in Eqs. (10), (12), and (14) as:

10 $${W}_{\Psi }^{H}\left(s, m, n\right)=\frac{1}{\sqrt{mn}}\sum_{x=0}^{m-1}\sum_{y=0}^{n-1}Img\left(x,y\right){\Psi }_{s, m,n}^{H}(x,y)$$

11 $$HL={\Psi }^{V}\left(x,y\right)=\varnothing (x)\Psi (y)$$

12 $${W}_{\Psi }^{V}\left(s, m, n\right)=\frac{1}{\sqrt{mn}}\sum_{x=0}^{m-1}\sum_{y=0}^{n-1}Img\left(x,y\right){\Psi }_{s, m,n}^{V}(x,y)$$

13 $$HH={\Psi }^{D}\left(x,y\right)=\Psi (x)\Psi (y)$$

14 $${W}_{\Psi }^{D}\left(s, m, n\right)=\frac{1}{\sqrt{mn}}\sum_{x=0}^{m-1}\sum_{y=0}^{n-1}Img\left(x,y\right){\Psi }_{s, m,n}^{D}(x,y)$$

**Figure 3:**
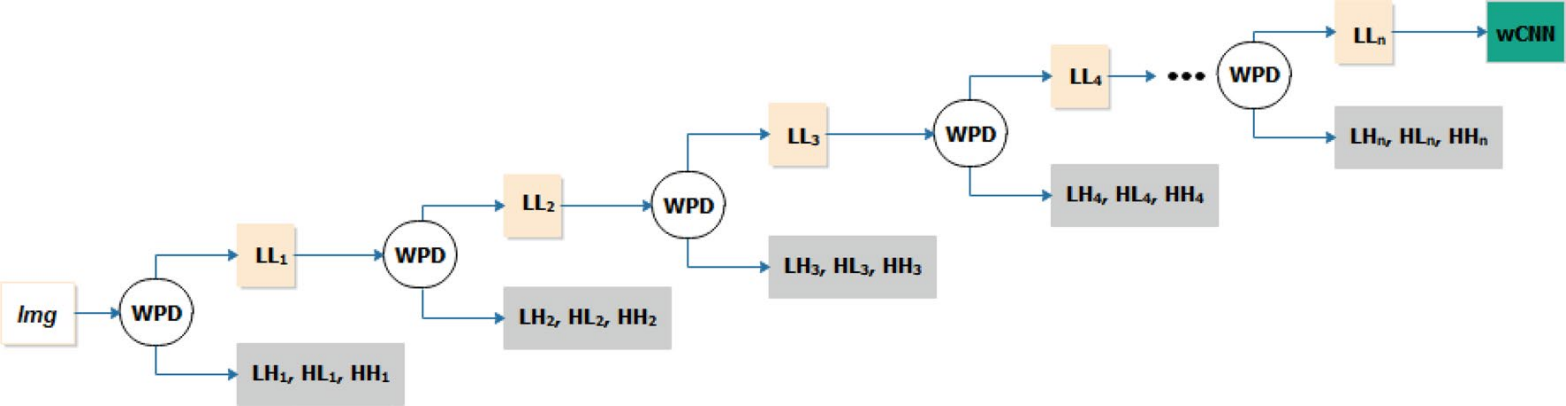
Illustration of subbands of coefficients formed after n-level application of wavelet decomposition function.

Figure 3 illustrates using a hierarchical representation of how *Img* is decomposed using the wavelet functions described previously. To obtain a good resolution of images for our CNN architecture, we allowed the wavelet decomposition function to decompose the original image to the highest N level of *n*.

The two-dimensional (2D) wavelet multilevel decomposition function was applied to our images, which were enhanced using the CLAHE technique. A decomposition level *n* was used, where n > 1 was obtained by computing the decomposition wavelet transform maximum level. The resulting low pass coefficient from the *n-level* decomposition was supplied to the wCNN architecture. This allows for obtaining the approximate features of the image samples at their best resolution and then used for the feature extraction and classification procedure in the CNN model.

### The wCNN architecture

**Figure 4:**
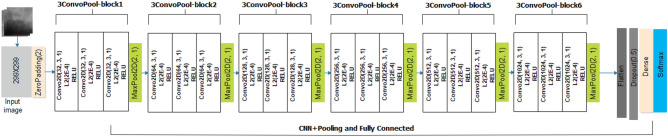
The architecture of the proposed CNN model for characterization of abnormalities in breast images.

The design of the CNN architecture assumes a twofold approach involving the traditional CNN architecture, which uses rectified linear units (RELU) as the activation function in the units of the convolutional layers, and the wavelet CNN (wCNN), which uses a wavelet function to replace RELU. The CNN architecture of the proposed CNN in this study is shown in Fig. 4. The input is sized in the dimension of 299 × 299 for the grey-style image. A zero-padding operation is first applied on the input before being passed into the CNN layers. There are six (6) blocks of convolutional operations, with each block comprising three layers of convolution operation followed by a pooling layer. In each convolutional layer, the L2 regularizer is applied with a factor of 0.0002. Additionally, we applied a 3 × 3 filter for each unit in the convolutional layers. The filter count assumes a $filte{r}_{count}= {2}^{n}$, where $5 \ge n \le 9$. The activation layer applied for the probability map in the output is the softmax function. This allows for categorization, which is patterned after the multiclass nature of the classification task. We investigated the performance of the CNN model using SGD and Adam optimizers. Meanwhile, a dropout layer with a drop rate of 0.5 is applied after the flattened layer.

**Figure 5:**
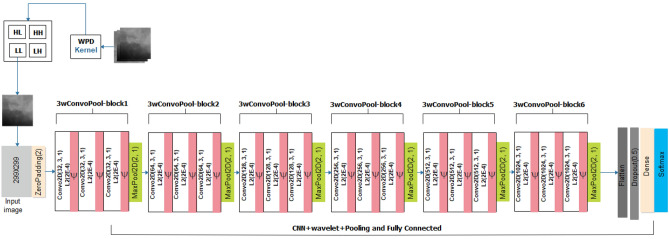
The proposed wCNN model architecture for characterization abnormalities in breast images using features from the WPD operation and wavelet function in the convolutional layers.

On the other hand, we show the architecture of the wavelet CNN (wCNN) in Fig. 5 to describe its configuration. It assumes a similar architectural configuration compared with the vanilla CNN described earlier. However, the input supplied to it are features extracted from the Wavelet packet decomposition (WPD) function described in the image preprocessing section. The architecture also uses a wavelet function proposed to replace the RELU function used in the CNN architecture. We maintain that the convolutional blocks and their corresponding filter size and count are the same.

Basically, the mathematics of convolutional network for a two-dimensional input image performs the convolved with filter *f* as seen in Eq. (15), a summation in Eq. (16), and then application of activation function, an example of which is shown in Eq. (17).

15 $${z}_{1}=x*f$$

16 $$x=\sum_{i=0}^{n}\sum_{j=0}^{m}{(z}_{i,j} . {w}_{i, j})+b$$

17 $$f\left(x\right)=relu\left(x\right)=\mathrm{max}(0, x)$$

This study replaces (17) with a wavelet equation in (18) to achieve the activation function for both the convolutional and fully connected layers.

18 $$\Psi \left(x\right)=\mathrm{cos}\left(beta . x\right){e}^{-(\frac{{X}^{2}}{2})}$$

where *beta* assumes a value of 0.5 and *x* the input image. The following demonstrates the applicability of the proposed wavelet function in a neuron or unit of a convolutional layer. Given an input image, *X*, which is the output from the wavelet decomposition function in the image preprocessing stage, we obtain the approximate coefficient of *X* and supply it as input to the CNN architecture. Then, we zero-pad the input as shown in Eq. (19), assuming our input is of size m = 3, n = 3:

19 $$X=\begin{array}{ccc}{x}_{11}& {x}_{12}& {x}_{13}\\ {x}_{21}& {x}_{22}& {x}_{23}\\ {x}_{31}& {x}_{32}& {x}_{33}\end{array} \mathrm{Zeropad}\left(X\right)= \begin{array}{cc}0& 0\\ 0& {x}_{11}\\ 0& {x}_{21}\end{array} \begin{array}{ccc}0& 0& 0\\ {x}_{12}& {x}_{13}& 0\\ {x}_{22}& {x}_{23}& 0\end{array}$$

$$\begin{array}{ccc}0 & {x}_{31}& {x}_{32}\end{array} \begin{array}{cc}{x}_{33}& 0\end{array}$$

$$\begin{array}{ccc}0 & 0& 0\end{array} \begin{array}{cc}0& 0\end{array}$$

After the zero-padding operation, the resulting input *X* is then passed into the units of the first convolutional layer so that the convolution operation is applied as described earlier. Then, the summation operation for that unit is computed considering the weights and bias values. The outcome of these operations is then passed into the proposed wavelet function to perform the activation operation. This is summarized in Eq. (20):

20 $$\Psi \left(X\right)=\mathrm{cos}\left(1.0 . X\right){e}^{-(\frac{{X}^{2}}{2})}$$

Equation (20) describes the forward pass in the CNN network. For backpropagation, we show that the derivative of (20) is obtained, then the derivative of (15) and (16), respectively. This is summarized in Eq. (21):

21 $$\frac{\partial x}{\partial f}=\frac{\partial (x . f)}{\partial {z}_{1}} . \frac{\partial (\sum_{i=0}^{n}\sum_{j=0}^{m}{((x*f)}_{i,j} . {w}_{i, j})+b)}{\partial x} . \frac{\partial (\mathrm{cos}\left(1.0 . x\right){e}^{-(\frac{{x}^{2}}{2})})}{\partial\Psi }$$

The outcome of this derivative yields the error resulting from the forward pass on the input compared with the actual value. The experimentation of the CNN architectures described in this section will be demonstrated in the following by applying the activation functions described in Eqs. (17) and (20) for the CNN and wCNN, respectively.

## Experimentation

In this section, details on the image datasets used for the experimentation are given, and the performance of the image preprocessing operations are evaluated and discussed. Additionally, the parameters and hyperparameters used for training the CNN and wCNN architectures are presented. Meanwhile, the system configuration of the computational environment is detailed to reinforce other parameters that shall be supplied to support the reproducibility of the experiment. Finally, a list of some evaluation metrics is discussed and applied for the performance evaluation of the two networks.

### Configuration of the experimental environment

Training and testing were experimented with using the TensorFlow library and dependent libraries using Python 3.7.3. The computational environment consists of an Intel (R) Core i5-7500 CPU 3.40 GHz, 3.41 GHz; RAM of 16 GB; 64-bit Windows 10 OS.

### Benchmark datasets for experimentation

**Figure 6:**
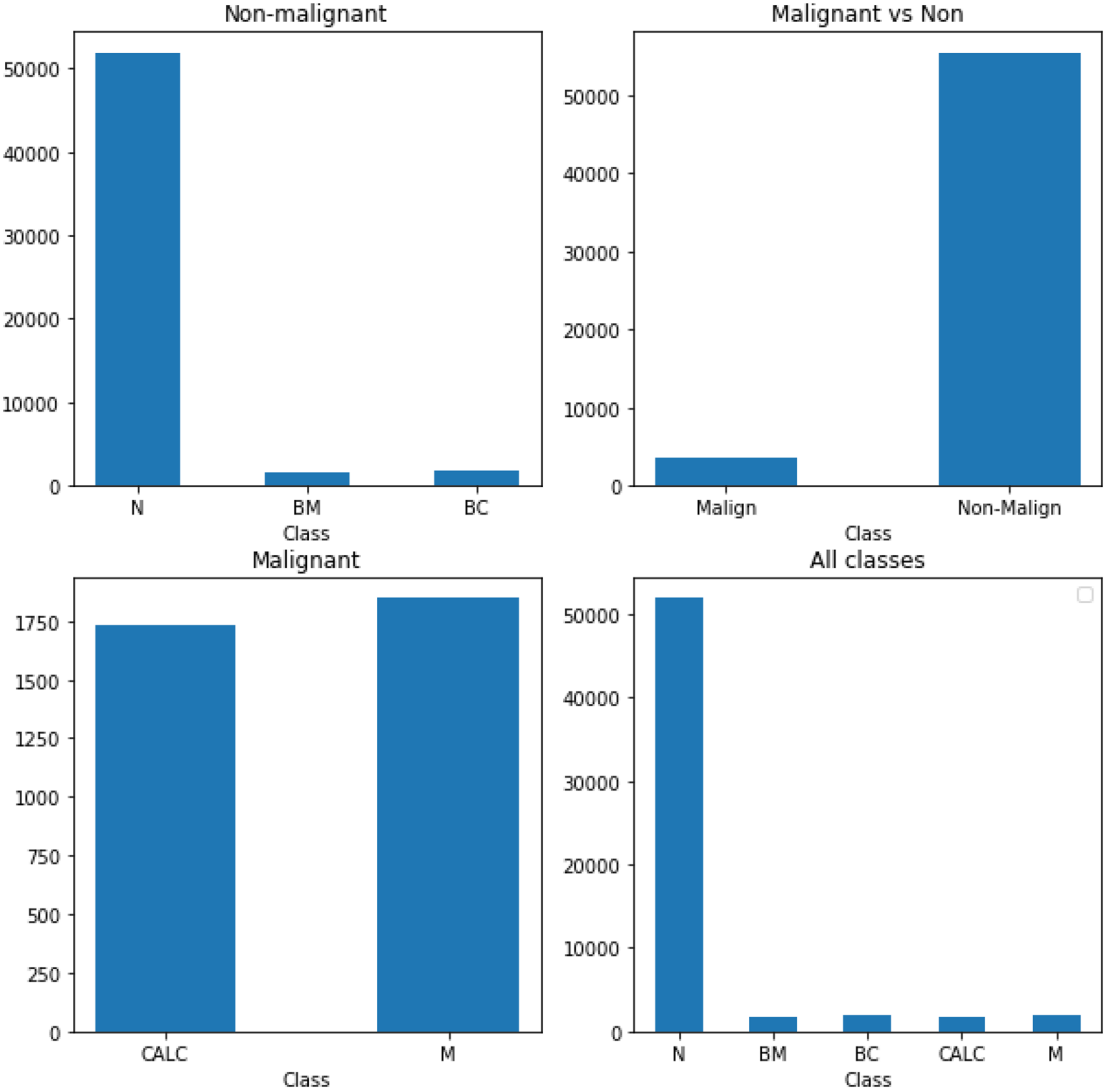
A graphical illustration of the distribution of image samples from the DDSM + CBIS and MIAS datasets. The distribution was graphed using the malignant and nonmalignant cases and further grouped according to the labelling as follows: *BC* benign calcification, *BM* benign mass, *CALC* calcification, *M* mass.

The Mammographic Image Analysis Society (MIAS)^55^ and Curated Breast Imaging Subset (CBIS) of the Digital Database for Screening Mammography (DDSM + CBIS)^38^ datasets were used for experimental purposes in this study. The two datasets contain samples with normal and abnormal observations. For instance, abnormal samples were classified as either benign or malignant. Those with benign abnormalities were either defective by calcification or the presence of mass abnormalities. Similarly, those with malignant cases had either calcification or the presence of a mass, as abnormalities were reported. In Table 3, a summary of the statistics of the datasets is listed, and further description is given. Figure 6 presents a graphical illustration of the distribution of image samples from the DDSM + CBIS and MIAS datasets.

**Table 3:** List of mammography databases applied for experimentation.

Database image information			Class Distribution					Source description
Dataset	Image views	No. samples	N	BC	BM	CALC	M	Description
MIAS	MLO	3075	2718	45	159	36	117	From reduced 200-micron pixel of sizes 1024 × 1024. We obtained 3075 ROI-based extractions of 299 × 299 sizes stored as NumPy files
DDSM + CBIS	MLO and CC	55,904	48,735	1884	1709	1731	1844	14% are positive and the remaining 86% negative. We used ROIs-based image size 299 × 299 and stored as tfrecords files

*MLO* mediolateral oblique view, *CC* craniocaudal, *N* normal, *BC* benign calcification, *BM* benign mass, *CALC* calcification, and M- mass.

**Figure 7:**
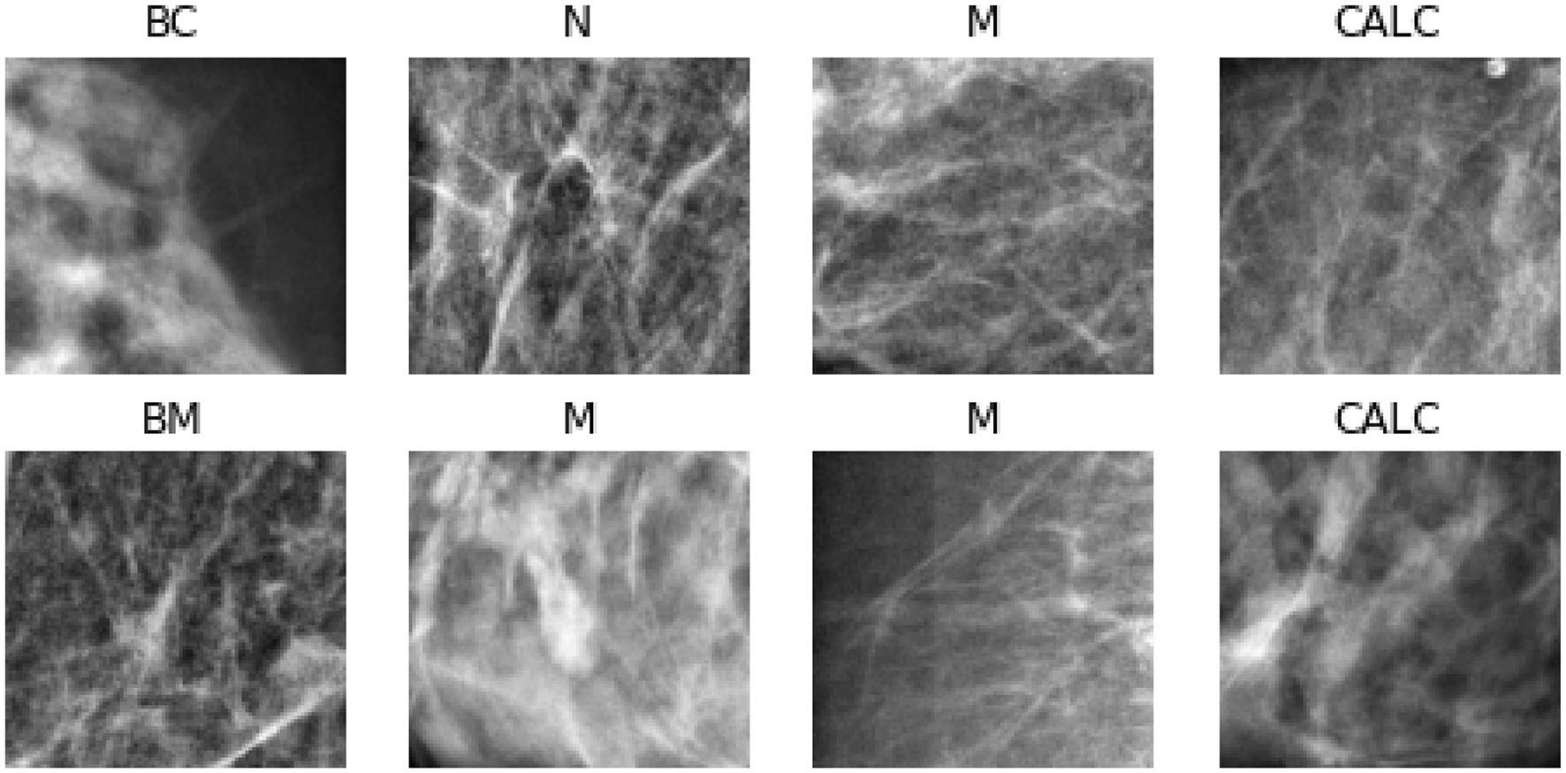
Samples of images with abnormalities as collected from the DDSM + CBIS and MIAS datasets with labels denoted as *BC* benign calcification, *BM* benign mass, *CALC* calcification, *M* mass.

To illustrate the distribution of samples in the MIAS and DDSM + CBIS datasets, we plot the distribution of the samples across classes and further divide samples into malignant and nonmalignant. In Fig. 6, a comparison of the distribution of classes of samples into malignant and nonmalignant is shown. Additionally, separate graphs for the distribution of classes in the malignant and nonmalignant cases are displayed. Finally, we show a graph for the distribution of all classes. These graphs allow for understanding the spread of samples across the five (5) classes. Samples with normal labels are seen to dominate the distribution, while in the classification between malignant and nonmalignant, the latter dominates the former. We observed a fair distribution of samples across the calcification and mass abnormalities among the malignant lesions. However, those with mass labels were slightly more abundant than those with calcification. Figure 7 shows some samples randomly drawn from the combined databases. The labels are interpreted thus: normal (N), (BC) benign calcification, benign mass (BM), calcification (CALC), and mass (M). In the experimentation, samples from the DDSM + CBIS database were used for training and evaluation, while those from the MIAS were used for testing. This allows for a fair evaluation of the proposed CNN and wCNN architectures since the MIAS samples are different from the DDSM + CBIS.

### GAN image augmentation: sample generated

The image samples shown in the last section from the two databases consist of abnormalities and normal cases. Those in the abnormalities category with malignancy were further categorized into mass and calcification abnormalities. These two classes of abnormalities often dominate most publicly available databases and are reported to be commonly diagnosed. We, however, note that other abnormalities, such as asymmetry and architectural distortion, have been shown to be delicate, subtle and fatal when overlooked^3^. As a result, an already trained GAN model was applied to generate image samples in these two categories. The details of the GAN model are presented in “Methodology”.

**Figure 8:**
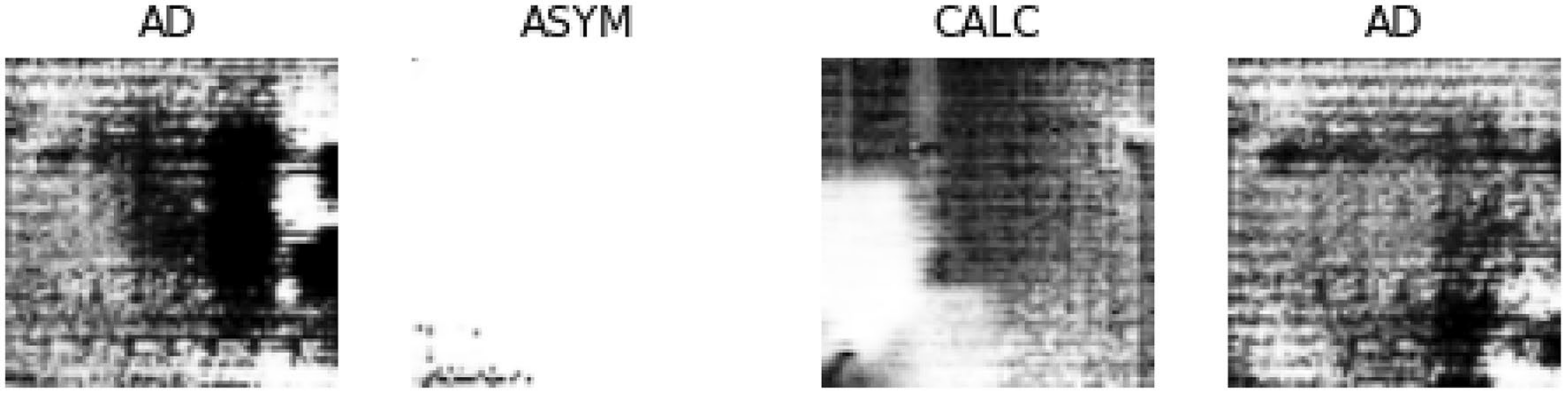
Samples of images generated using a GAN model for augmenting samples available from DDSM + CBIS and MIAS datasets. The samples generated images with architectural distortion (AD), asymmetry (ASYM) and calcification (CALC).

In Fig. 8, we show some samples of images generated using the GAN model described in “Methodology”. Images belonging to architectural distortion (AD), asymmetry (ASYM) and calcification (CALC) were synthesized to augment missing samples from classes AD and ASYM and to augment the quantity of those in CALC. With this synthetization process, sufficient data have been sourced for the experimentation procedure to allow our model to generalize well and overcome overfitting. These samples were added to those used for training and evaluation while keeping the MIAS images for testing the fully trained CNN and wCNN models.

### Implementation of image preprocessing

**Figure 9:**
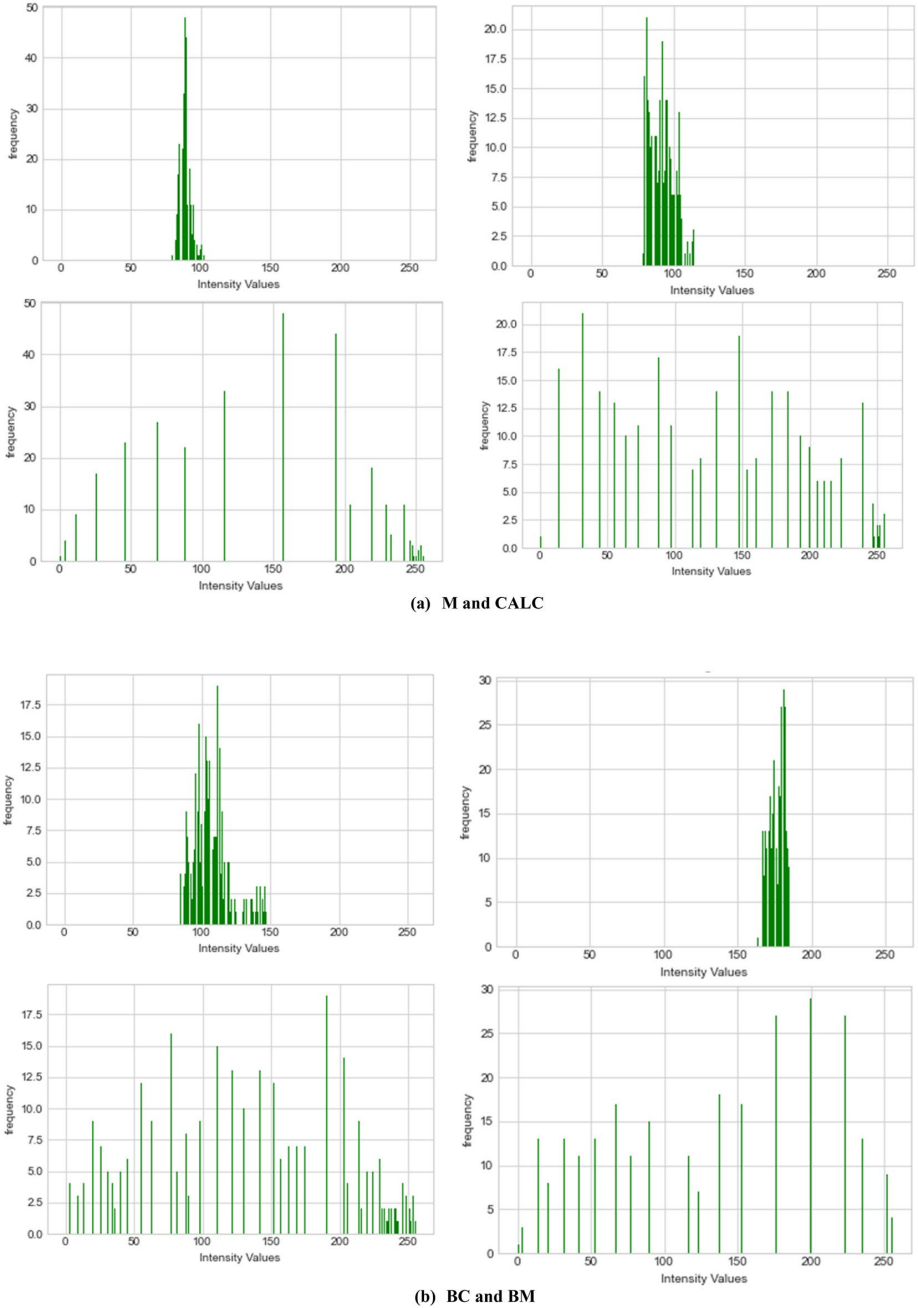
Computation and graphing of the histogram with the corresponding equalized histogram for the image samples used for the experimentation preprocessed using CLAHE. (**a**) M and CALC abnormalities and (**b**) shows BC and BM abnormalities.

Image preprocessing techniques were applied to all samples drawn from DDSM + CBIS, MIAS and those generated using the GAN model. First, to understand the need for improvement in the images, we plotted their corresponding histogram to investigate how pixel values are distributed. This understanding led to the use of image enhancement techniques to equalize the distribution of pixel values in the histogram. In Fig. 9, the first row of the figure shows the histogram for image samples with M, CALC, BC, and BM abnormalities. In row two, their corresponding equalized histograms were plotted to compare the improvement achieved easily.

**Figure 10:**
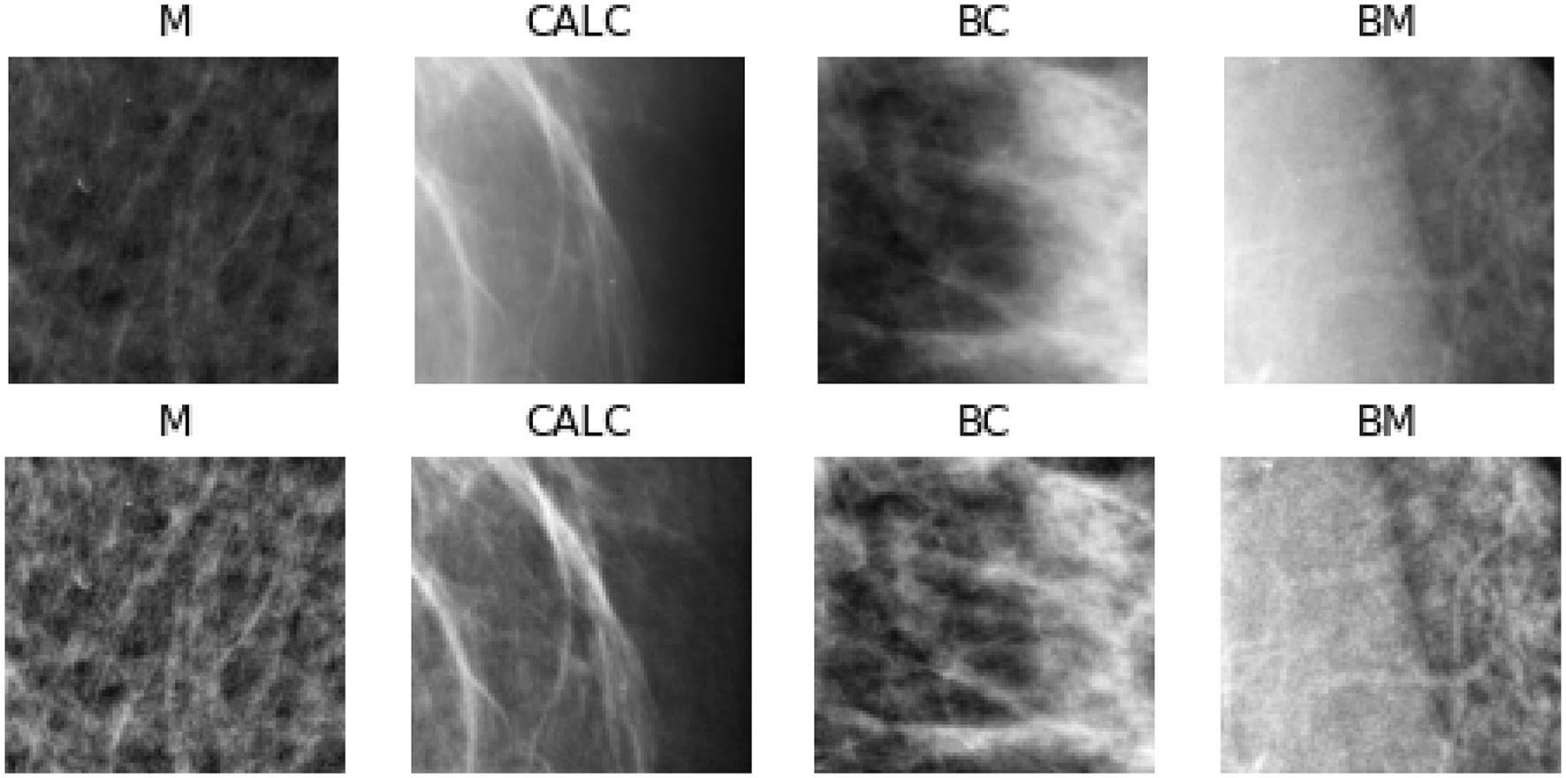
Samples of images to demonstrate the improvement resulting from preprocessing all image inputs using the enhancement technique CLAHE.

In Fig. 10, the images of the histograms shown in Fig. 9 are listed and their corresponding enhancement. As mentioned in “Methodology”, the CLAHE technique was used for the improvement of the samples. The first row of Fig. 9 shows the raw image, which was not preprocessed, while the second row shows the corresponding image enhanced using the CLAHE method. Clearly, we see that the preprocessing method successfully improved the images with some blurriness eliminated, yielding quality images.

**Figure 11:**
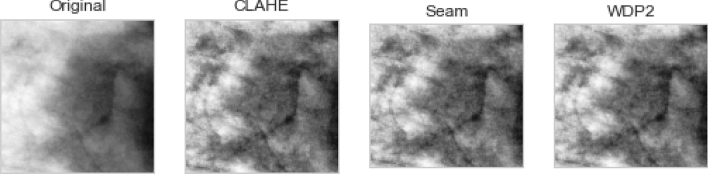
A progressive display of the preprocessing methods applied to a sample image from the data sources. The original image, its corresponding CLAHE-operated version, its corresponding seam-carving-operated version, and its wavelet-operated version.

**Figure 12:**
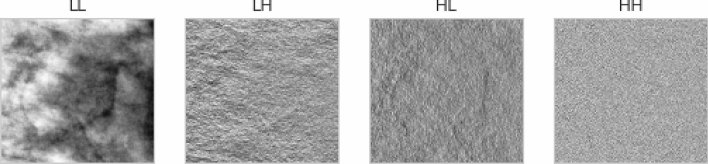
Comparison of the approximate coefficient (LL) and the detailed subbands (*LH* horizontal, *HL* vertical, *HH* diagonal), also known as high pass.

The preprocessed image samples were further supplied as input to the seam carving technique to remove the least significant pixels. The outcome of the seam carved method was then supplied to the wavelet decomposition function for extraction of relevant features required for the convolutional operation. Figure 11 shows the impact of applying these techniques as an arbitrary sample is used for this comparison. The original image is shown, while the corresponding version applied to CLAHE is seen to be improved. Furthermore, the seam carving operation showed that some pixels were eliminated from the outcome of the CLAHE operation. Finally, the image resulting from applying the wavelet function (WPDP2) on the outcome of seam carving is also shown. We note that the approximate coefficient (LL) image from the wavelet function is chosen for use here, as shown in Fig. 12.

As mentioned in “Methodology”, the LL version of each image containing features supportive of the feature detection task was used for the CNN and wCNN models during the experimentation phase.

### CNN configuration and training parameters

The CNN and wCNN architectures were tested for twenty (20) epochs, and validation was also performed during the training. The Adam optimization algorithm was used for training the two models, and we experimented using learning rates of 1e−05 and 1e−06. Since all samples used for the experiment are grayscale, the image was sized 299 × 299 so that the dimensions of all inputs were 299, 299, and 1. A batch size of 64 was used during training for passing image samples into the convolutional layers.

### Evaluation metrics

There are several evaluation metrics for comparing the performance of learning models and classifiers. Some of these metrics are the confusion matrix, accuracy, receiver operating characteristic (ROC) curve, area under the ROC curve (AUC), precision, recall, precision, specificity, Matthew correlation coefficient (MCC) for the binary classifier, and F1 score. In this paper, we evaluated the following: accuracy, the area under the ROC curve (AUC), specificity, sensitivity, precision, F1 score, false-positive rate (FPR), and recall. In this study, some of these metrics were selected to evaluate the performance of the two major deep learning architectures. The following are the metrics applied for result evaluation and the justification for their selection:

#### Accuracy

Accuracy is a widely used metric in most classification and deep learning models. It allows us to evaluate whether we have trained our model well enough to generalize to new samples. This model evaluation using accuracy is performed across all classes in our datasets. We, however, note that where class imbalance exists, accuracy may not present the true performance of our model; hence, other metrics are considered in this study. We measure accuracy using Eq. (22).

22 $$Accuracy=\frac{TP+TN}{(TP+TN+FP+FN)} $$

#### Specificity

The specificity metric is used to compute the total number of actual negative cases (normal and benign) in our datasets, which our proposed model discovered to be truly negative. In Eq. (23), we show how to compute specificity.

23 $$Specificity=\frac{TN}{(TN+FP)}$$

#### Sensitivity

This metric allows for computing the number of actual positive cases in our datasets that were predicted as true positives. The equation for computing the sensitivity metric is given in Eq. (24)

24 $$Specificity=\frac{TP}{(TP+FN)} $$

#### Precision

To eliminate the presence of false positives and ensure that our model correctly classifies negative cases as negative and positive cases as positive, we use the precision metric to evaluate our models. The precision metric supports the ability to determine how correctly our model predicts positive cases. The equation is given in (25)

25 $$Precision=\frac{TP}{\left(TP+FP\right)} $$

#### F1 score

The F1-score is computed using a combination of recall and precision. This then allows for using the metric as the weighted average of the two underlying metrics. The best performance of our model as it relates to the F1-score metric will be indicated by a value that tends towards 1.0, while a value closer to 0.0 demonstrates poor performance. The equation is given in (26).

26 $$F1-Measure=\frac{\left(2\times Precision\times Recall\right)}{\left(Precision+Recall\right)} $$

#### Recall

To measure the proposed models' ability to pick out positive samples from the data source used for the experiment, we evaluate them using recall metrics. A higher value obtained for recall implies how accurately our model can identify abnormalities in the datasets. The equation for computing the metric is given in (27).

27 $$Recall=\frac{TP}{(TP+FN)} $$

In the following section, the metrics discussed here are applied to all experiments carried out for a fair comparative analysis.

## Results and discussion

The last section details the configuration leading to experimentation of the concept described in this paper. This section presents a listing of the results obtained in the experiments conducted. Comparative analysis of the results obtained for all experiments was carried out, and findings from the performance of each experiment and case and its performance with respect to the experimental setup are discussed. The section then concludes by highlighting the relevance of the proposed approach in the models' applicability to breast cancer detection.

Four (4) major experiments were conducted as follows: experimenting with the proposed CNN with normal samples, experimenting with the proposed CNN with CLAHE-WPD-operated samples, experimenting with the proposed wavelet-CNN with CLAHE-WPD-operated samples, and wavelet-CNN with CLAHE-WPD-GAN samples. We also investigated the impact of applying the GAN model for image augmentation purposes. The results obtained follows in the subsections.

**Figure 13:**
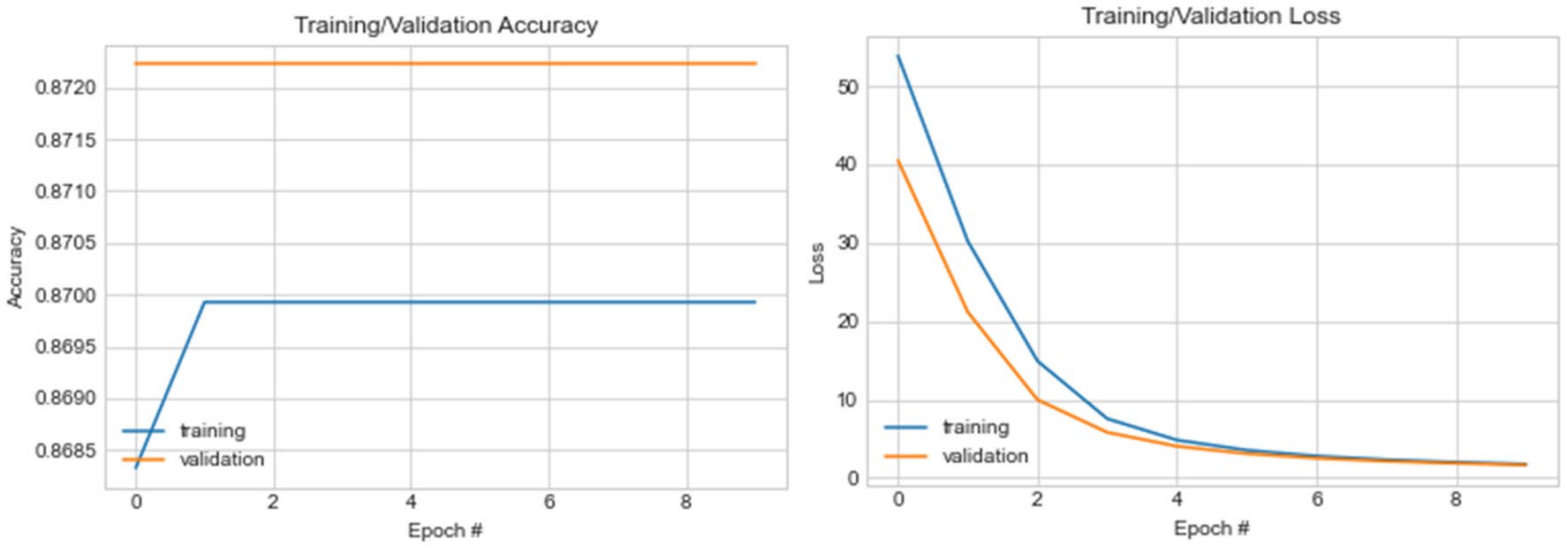
A graphical illustration of accuracy and loss value distribution for ten (10) epochs on the CNN model using normal samples.

The basic CNN architecture experiment shows that the training accuracy obtained ranges from approximately 0.86832, while the validation accuracy steadied at approximately 0.87222. The loss value for the training phase dropped from 53.86 at the first epoch to 1.77 at the tenth epoch. For the validation phase, we observed that the loss value dropped from 40.52 to 1.66. These patterns in the change of values for both accuracy and loss values demonstrate a good performance by the CNN architecture in detecting and classifying features. In Fig. 13, a graph illustrating the plot of the values obtained in both the training and validation phases is shown.

**Figure 14:**
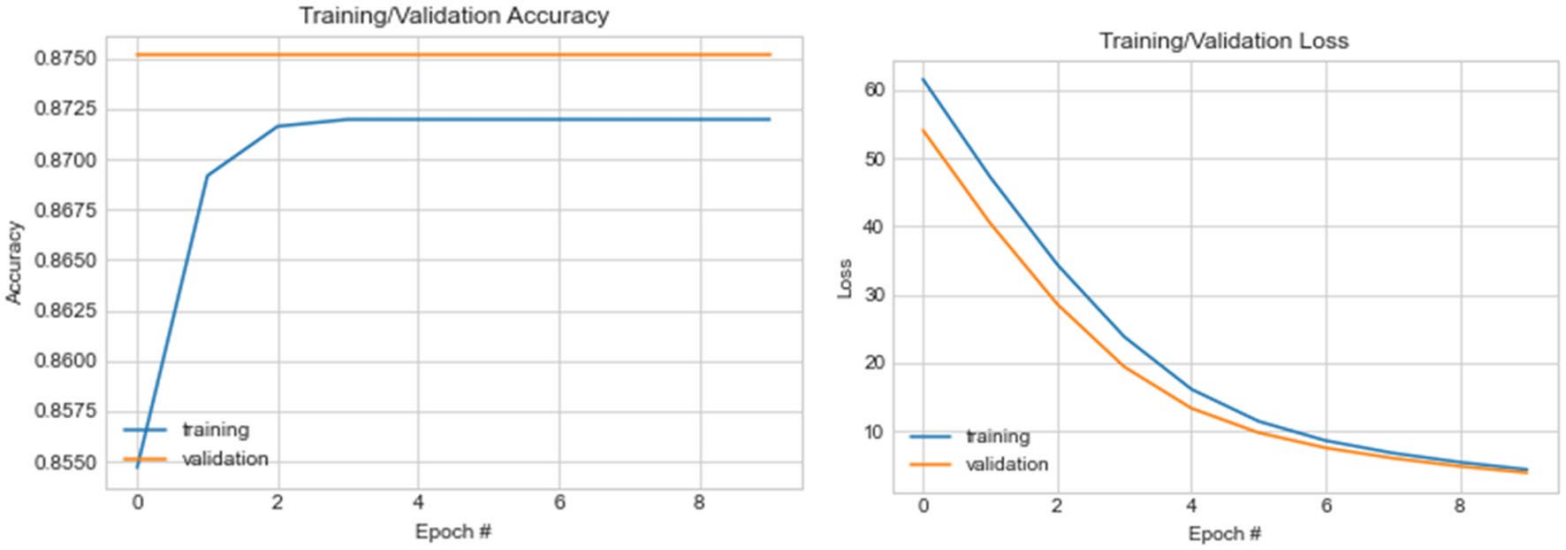
A graphical illustration of accuracy and loss value distribution for ten (10) epochs on the wavelet-CNN model using CLAHE samples.

Similarly, we trained the wavelet-CNN architecture under the same configuration with ten (10) training epochs, which uses the samples preprocessed using the CLAHE method. We observed performance improvement for training accuracy, loss values, and even validation. For instance, we see that accuracy rose to 0.8716 compared with what is obtainable with the basic CNN. This indicates performance enhancement resulting from the proposed wavelet transformation function applied to the CNN structure. The same is seen for the validation accuracy, which yielded 0.87514, an improvement compared with the basic CNN structure. Relatively similar loss values are seen in basic CNN and that of wavelet-CNN. The implication is that the wavelet transformation function competes with those popularly used in the literature. The graphing of the values for both the accuracy and loss are shown in Fig. 14.

**Figure 15:**
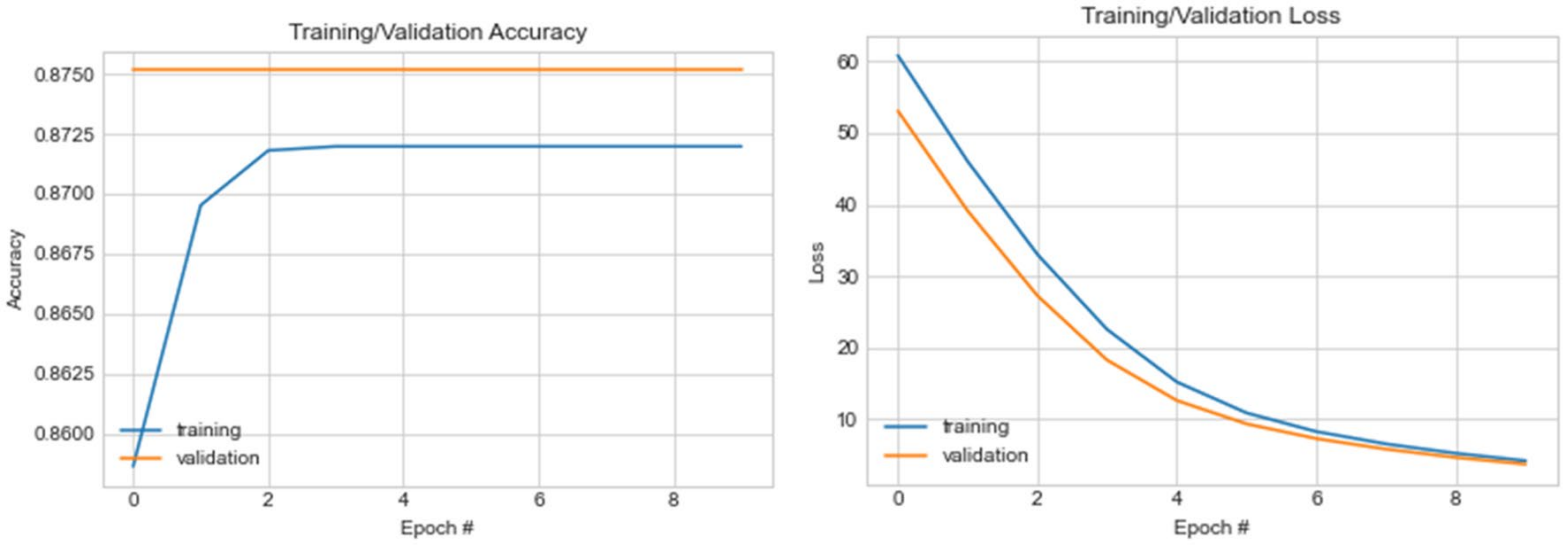
A graphical illustration of accuracy and loss value distribution for ten (10) epochs on the wavelet-CNN model using CLAHE + WPD samples.

Now, we investigate the performance combining the decomposing wavelet function and the wavelet transformation function to compare the output with that previously discussed. Interestingly, while the classification accuracy is sustained, we observed a slight improvement in the learning rate and returned loss function values. For instance, in the case of wavelet-CNN and normal samples with CLAHE operations, the loss values for the first and last epochs are 61.60 and 60.85, respectively. On the other hand, in the case of the same wavelet-CNN and seam carving with wavelet decomposition image samples, CLAHE operations, the loss values for the first and last epoch are 60.85 and 4.15, respectively. This implies that the wavelet transform function sustains classification accuracy, while the quality of image samples supplied as input contributes to the loss values obtained. In Fig. 15, the graph showing the plots for the accuracy and loss values obtained for ten (10) epochs are shown.

**Figure 16:**
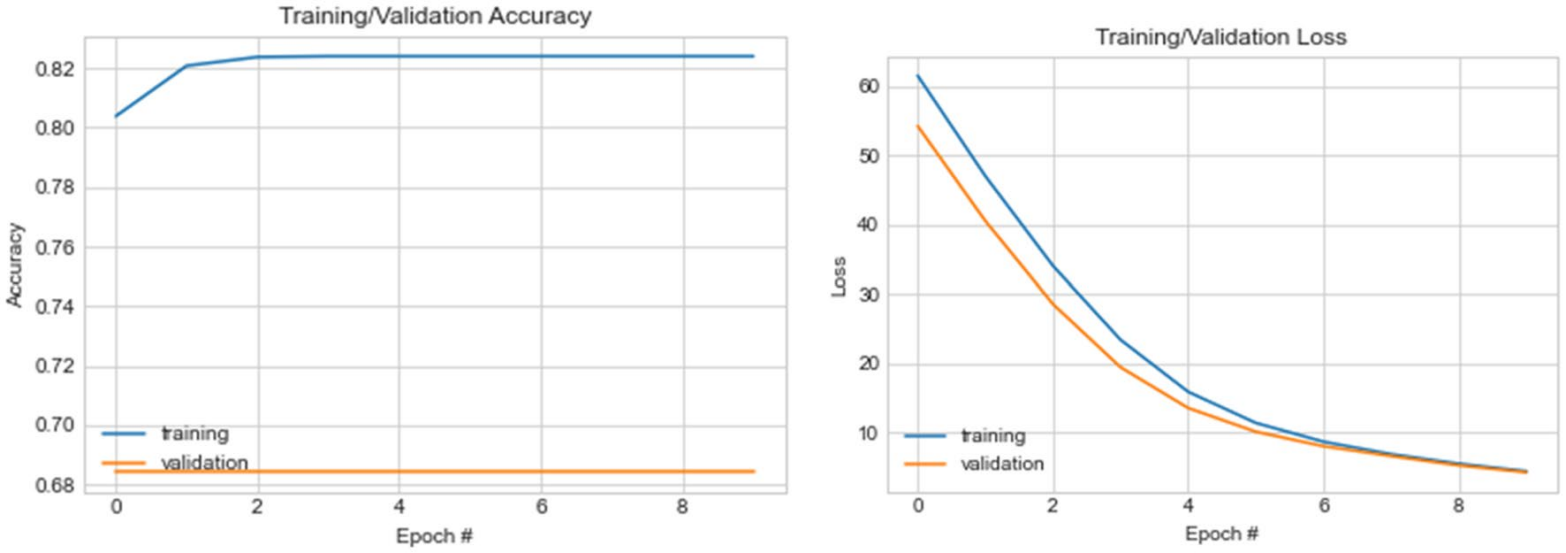
A graphical illustration of accuracy and loss value distribution for ten (10) epochs on the wavelet-CNN + CLAHE + WPD model using real and GAN synthesized samples.

Having confirmed that input samples determine the loss values during training and evaluation, we augment our datasets using samples synthesized using the GAN model described in the previous section. This leads to the outcome of the fourth experiment shown in Fig. 16. In this experiment, we investigate what performance improvement is obtainable when the synthesized samples are subjected to wavelet-CNN and when combined with samples derived from seam carving with wavelet decomposition.

To demonstrate a comparison of the methods, we summarize the performance of the methods over the ten (10) epochs for all experiments performed and provide the outcome of the values obtained for both accuracy and loss. In Table 4, these values are listed and compared against each technique. The results obtained, as listed in the table, result from applying the DDSM + CBIS dataset on the training phase of all our experiments for the two models. The accuracy curves for most of the experimentations in the training phase rose from lower accuracy to higher values and stabilized around a reasonable level. This indicates that while the lose values drop signaling the fact that the model continues to improve its learning curve, the classification accuracy improved to attain stability.

**Table 4:** A summary of the comparison of performances of accuracy and loss values on CNN, wavelet-CNN on samples from CLAHE, WPD and GAN-based data augmentation techniques on the DDSM + CBIS benchmarked datasets used for training on last ten (10) epochs.

Epoch	CNN		Wavelet-CNN + CLAHE		Wavelet-CNN + CLAHE + WPD		Wavelet-CNN + CLAHE + WPD + GAN	
	Accuracy	Loss function	Accuracy	Loss function	Accuracy	Loss function	Accuracy	Loss function
1	0.8683	53.8682	0.8547	53.9556	0.8586	60.8583	0.8038	61.5979
2	0.8699	30.2180	0.8692	30.5985	0.8695	46.0458	0.8208	47.0644
3	0.8699	14.9185	0.8716	15.3767	0.8718	33.0377	0.8237	34.0883
4	0.8699	7.5588	0.8720	7.9842	0.8720	22.5305	0.8240	23.4251
5	0.8699	4.8278	0.8720	5.2136	0.8720	15.1756	0.8240	15.8924
6	0.8699	3.5453	0.8720	3.9151	0.8720	10.8479	0.8240	11.3898
7	0.8699	2.7984	0.8720	3.1491	0.8720	8.2372	0.8240	8.6747
8	0.8699	2.3312	0.8720	2.6543	0.8720	6.5132	0.8240	6.8728
9	0.8699	2.0073	0.8720	2.3020	0.8720	5.2032	0.8240	5.4966
10	0.8699	1.7730	0.8720	2.0363	0.8720	4.1580	0.8240	4.4030

The summary of performances of CNN, Wavelet-CNN + CLAHE, Wavelet-CNN + CLAHE + WPD, and Wavelet-CNN + CLAHE + WPD + GAN models as listed in the table reveals the marginal difference existing among them. This is particularly obvious in the classification accuracy for the four models experimented with. In the cases of wavelet-CNN + CLAHE and wavelet-CNN + CLAHE + WPD, there appeared to be a marginal difference in their classification accuracy. This is supported by the fact that the wavelet transformation is applied to both cases. However, where the wavelet transform function is not applied, we see a drop in classification accuracy. Interestingly, the loss values obtained for both Wavelet-CNN + CLAHE and Wavelet-CNN + CLAHE + WPD progressively dropped as their accuracy values improved. The two methods, Wavelet-CNN + CLAHE and Wavelet-CNN + CLAHE + WPD, use the proposed wavelet function, hence the competitive result obtained in both experiments. This confirmed that using the wavelet function improved the CNN model compared with what is obtained using the RELU activation function.

After completely training the two models CNN and wavelet-CNN, we applied the MIAS dataset for the testing phase. This became necessary to demonstrate fairness for the testing procedure of the proposed models. We considered that since the MIAS samples are from a different dataset from that of DDSM + CBIS used for training, it will help to validate the ability of the CNN and wavelet-CNN models to generalize well. Using the metrics discussed in “Experimentation”, the results in Table 5 are presented to compare the techniques proposed in this study.

**Table 5:** A summary of the comparison of the performances of CNN and wavelet-CNN on samples from CLAHE-, WPD- and GAN-based data augmentation techniques on the MIAS benchmarked datasets used for testing.

Model	Accuracy	Specificity	Precision	F1-score	Recall
CNN	0.8751	1.0	0.8751	0.8751	0.8751
Wavelet-CNN + CLAHE	0.9990	1.0	0.9990	0.9990	0.9990
Wavelet-CNN + CLAHE + WPD	0.9990	1.0	0.9990	0.9990	0.9990
Wavelet-CNN + GAN + WPD	0.9990	1.0	0.9990	0.9990	0.9990

Comparison of accuracy, specificity, precision, F1-score and recall metrics as obtained in the models revealed interesting performances. The trained models of wavelet-CNN + CLAHE and wavelet-CNN + CLAHE + WPD, when used for prediction on both MIAS and fragments of DDSM + CBIS datasets, showed similar performance. Their accuracy precision, F1-score and recall values stood at approximately 0.99 while specificity was 1.0. compared with the performance of the trained basic CNN model, which obtained accuracy precision, F1-score and recalls values stood at approximately 0.87 while specificity is 1.0, there is an improvement in performance due to the method proposed in this study. These prediction performances with the trained models demonstrate that using the wavelet transform function to extract features in the sample images in digital mammography is relevant.

**Figure 17:**
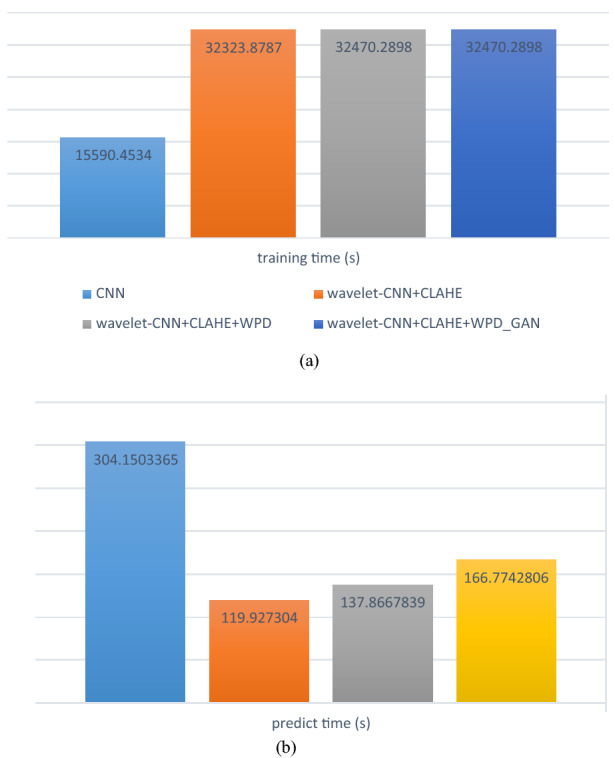
A comparison of computational time for (**a**) training and (**b**) prediction of basic CNN, wavelet-CNN on samples from CLAHE, WPD and GAN-based data augmentation techniques.

Meanwhile, we computed the training and prediction time for all four models experimented on in this study. In Fig. 17, the graphed results show that the training time for the basic CNN was lower than those of the proposed hybrid methods, so its prediction time was unattractively high. The other three models are wavelet-CNN + CLAHE, wavelet-CNN + CLAHE + WPD, and wavelet-CNN + CLAHE + WPD with GAN samples. Using the wavelet transform function, we observed similar computational times. For instance, the training times for wavelet-CNN + CLAHE and wavelet-CNN + CLAHE + WPD were 3232.8787 and 32,470.2898, respectively. The computational times for predicting wavelet-CNN + CLAHE and wavelet-CNN + CLAHE + WPD were 119.9273 and 137.8667, respectively. Although there appeared to be some closeness in their demand for computational power, we noticed that models with wavelet decomposition and seam carving algorithms consumed more time during the prediction phase.

Further to comparative analysis of the performance of our models in the case of different experimentations carried out, we compared our proposed technique with those reported in the literature. This allows for justifying the relevance of the proposed approach compared with those that have shown state-of-the-art performance in recent studies. The results of these comparisons are outlined in Table 6.

**Table 6:** Comparison of the performance of the proposed CNN and wavelet-CNN methods with similar approaches and the same datasets.

Author reference	Method	Performance
^5^	CNN and data augmentation	Accuracy: 0.90
^56^	DeepCAD: multilayer deep-learning architecture	Accuracy:0.915, AUC:0.91, sensitivity: 0.915, specificity: 0.842
^30^	DCNN: AlexNet Fine-tuned to classify two classes instead of 1000 classes	Accuracy: 0.736, AUC:0.94
^57^	VGGNet and ResNet	Accuracy: 0.925
^37^	Ensemble of convolutional neural networks for classification of breast microcalcification	Recall: 0.94, precision of 0.95
^46^	CNN-DW and CNN-CT with an augmented data set	Accuracy: 0.8374
This study	Wavelet-CNN-wavelet with augmented dataset using GAN	Accuracy:0.99, recall: 0.99, precision: 0.99, specificity: 1.0, F1-score: 0.99

The performance of the proposed method is compared with similar CNN models used for classification problems with digital mammography. Studies from 2016 to 2021 are listed in the table. Performance measures were computed using one or more of accuracy, recall, AUC, precision, sensitivity, specificity, and F1-score. In terms of accuracy, while the works of^5,56,57^, and^37^ yielded 0.90, 0.915, 0.736, and 0.925, respectively, the outcome of this study gave 0.9990. Interestingly, we compared the performance of our wavelet-CNN with a similar wavelet-CNN in^46^, and the result showed that our approach outperformed their own with a 16.16% increase. This again confirms the viability and relevance of the proposed wavelet transform function and the hybrid of seam carving with wavelet decomposition algorithms in this study. We see that classification accuracy is greatly enhanced compared with state-of-the-art models that have also applied wavelet transform functions. Additionally, in terms of loss values generated during the training, our method yielded a competitive performance with popular and state-of-the-art transform nonlinear functions. This study demonstrates the relevance of applying the wavelet function to extract discriminant features from digital mammography.

## Conclusion

In this paper, we presented a novel wavelet transform function to improve the structure of CNN architecture. This is intended to support detecting subtle and determinant features leading to the detection of abnormalities in digital mammography. Furthermore, image preprocessing was implemented using three methods to achieve an improved input sample. The methods applied are CLAHE for enhancement and seam carving and wavelet packet decomposition algorithms for feature enhancement. Meanwhile, to augment for insufficient image samples with architectural distortion, we applied a GAN model for synthetization of samples of that category. The combined methods were applied to DDSM + CBIS and MIAS datasets for experimentation. The results and discussion of the findings showed that the proposed method in this study improved performance compared with the basic CNN structure. In the future, we propose investigating the performance increment that will result from applying the wavelet transform function in the fully connected layers of the CNN architecture. In addition, the proposed method demonstrates that it can enhance the characterization of abnormalities in histopathological images in addressing the classification problem leading to the detection of breast cancer. The *beta* value used in the proposed wavelet function presents a performance tuning mechanism for increased accuracy. Therefore, we suggest as future research direction the investigative analysis of the impact of different values for the *beta* variable on the current model.

## Abbreviations

DL: Deep learning
CNN: Convolutional neural networks
DDSM + CBIS: Digital database for screening mammography + curated breast imaging subset
MNIST: Modified National Institute of Standards and Technology
ReLU: Rectified linear unit
CIFAR: Canadian Institute for Advanced Research
SVM: Support vector machine
ROIs: Regions of interests
CAD: Computer-aided detection
GAN: Generative adversarial network
2D-CNN: Two-dimensional CNN
COVID-19: Coronavirus disease-2019
NN: Neural network
FC: Fully connected
AUC: Area under curve
GURO: Korea University Guro Hospital
RetinaNet: Retina network
GooleNet: Google network
AlexNet: Ale network
FFDM: Full-field digital mammograms
InceptionV3: Inception version 3
TPR: True positive rate
FPI: Positives per image
ImageNet: Image network
FCN: Fully convolutional network
CRF: Conditional random fields
DDSM-BCRP: Digital Database for Screening Mammography -Breast Cancer Resistance Protein
MLO: Mediolateral-oblique
VGGNet: Visual geometry group network
ResNet: Residual network
MIAS: Mammographic Image Analysis Society
R-CNN: Region-based CNN
MLP: Multilayer perceptron
GlimpseNet: Glimpse network
CNN-DW: Convolutional neural network-discrete wavelet
CNN-CT: Convolutional neural network-curvelet transform
CLAHE: Contrast, limited adaptive histogram equalization
wCNN: Wavelet convolutional neural network
wCwNN: Wavelet convolutional wavelet neural network
CT: Computerized tomography
BPNN: Back-propagation neural network
NARX: Bonlinear autoregressive network with exogenous inputs
WNARX: Wavelet nonlinear autoregressive network with exogenous inputs
WNN: Wavelet neural network
WLSSVM: Wavelet-based least square support vector machine
ANN: Artificial neural network
mWDN: Wavelet decomposition network
RCF: Residual classification flow
mLSTM: Multi-frequency long short-term memory
LM: Levenberg–Marquardt
WPD: Wavelet packet decomposition
OS: Operating system
CPU: Central processing unit
RAM: Random access memory
CC: Craniocaudal
BC: Benign calcification
BM: Benign mass
M: Normal
CALC: Calcification
M: Mass
ASYM: Asymmetry
LL: Approximate coefficient
LH: Low pass horizontal (horizontal subbands)
HL: Horizontal low pass (vertical subbands)
HH: Horizontal high pass (diagnoal subbands)
WPDP2: Wavelet packet decomposition 2
ROC: Receiver operating characteristic
MCC: Matthew correlation coefficient
TP: True positive
FP: False positive
TN: True negative
FN: False negative
